# Tetanus

**Published:** 1829-02

**Authors:** 


### TETANUS.

*Case of Tetanus which proved fatal, showing a remarkable Alteration of the Medulla Spinalis.* By Dr. Poggi. (Hôpital d'Udine.)

A woman, aged forty-four, who was accustomed to remain for some time with her legs immersed in water, experienced, on the 8th of October, a great difficulty in moving the lower jaw, with a sense of rigidity of the muscles of the neck and stiffness of the limbs. She had previously, however, had attacks of pain after walking, which were considered to be rheumatic. On the 10th of October, when she entered the hospital, the tetanic contractions of the trunk and limbs were considerable, the trismus was violent. The latter was greatly relieved by a warm bath, but not so the other symptoms. The trunk was bent backwards, the arms and legs extended and stiff; breathing frequent and stertorous; abdominal muscles little contracted; bowels costive; urine small in quantity; pulse strong and frequent; skin hot and dry; intellectual faculties not in the least impaired, and sensation perfect in every part of the body. Thirst was great, and the tongue red and dry. The symptoms increased in intensity from the commencement until her decease, which took place five days after the attack.

The body was examined twenty-four hours after death. The brain was healthy. The spinal canal contained a larger quantity of serum than is usual, mixed with blood. The spinal arachnoid membrane presented no alteration. The pia mater showed signs of an increased action having existed, which was more apparent on the anterior than on the posterior aspect of the medulla. The medulla spinalis presented on its anterior half a multitude of granulous bodies, in size from a grain of millet to that of a lentil: it was very soft, and seemed entirely formed by the agglomeration of these globular bodies; its colour was a whitish yellow, and on its anterior surface, in different parts, small red points were to be seen. Its posterior half was perfectly healthy, and presented a remarkable difference to the anterior half. The white substance appeared to be the part that had undergone the greatest alteration, as the gray substance was apparently unaltered. The filaments of the anterior spinal nerves at their origin were sensibly diminished in thickness, and were of a yellowish colour, very soft and easily lacerated: several of them had, in their course, small tumors, such as those which were described on the anterior half of the medulla spinalis. The posterior spinal nerves, on the contrary, were healthy in appearance, and their volume and consistency were unaltered. The cavity of the chest contained a great quantity of bloody serum; the stomach and intestines exhibited slight traces of inflammation; the kidneys were of a brownish red colour, and gorged with blood; the bladder was much contracted, and contained but little urine.

This case, with others, will tend to prove that tetanus results from an affection of the spinal cord; although the singular alteration here met with seems to differ from the ordinary kind of lesions of this organ found after death, yet the appearance of great increased action of the pia mater, and the other phenomena mentioned above, show clearly that inflammation had primarily existed. And was not this the cause of the general affection, which had been improperly called rheumatism, of which the patient had complained for some time?

This case also shows the difference of the functions of the spinal nerves: sensation being perfect until the death of the patient, and the motive powers alone being affected. These phenomena will support the opinion that the anterior spinal nerves are those of motion, and the posterior those of sensation.

